# Nitric oxide favours tumour-promoting inflammation through mitochondria-dependent and -independent actions on macrophages

**DOI:** 10.1016/j.redox.2022.102350

**Published:** 2022-05-27

**Authors:** Daiana Drehmer, João Paulo Mesquita Luiz, Cesar Augusto Speck Hernandez, José Carlos Alves-Filho, Tracy Hussell, Paul Andrew Townsend, Salvador Moncada

**Affiliations:** a Division of Cancer Sciences, Manchester Cancer Research Centre, Faculty of Biology, Medicine and Health, University of Manchester, Manchester, UK; b Department of Pharmacology, Center of Research in Inflammatory Diseases (CRID), Ribeirão Preto Medical School, University of São Paulo, São Paulo, Brazil; c The Lydia Becker Institute for Immunology and Inflammation, University of Manchester, Manchester, UK

## Abstract

Production of nitric oxide (NO) has been demonstrated in several malignancies, however its role remains not fully understood, specifically in relation to the metabolic and functional implications that it may have on immune cells participating in tumorigenesis. Here, we show that inducible NO synthase (iNOS) is expressed in cancers of the colon and the prostate, mainly by tumour cells, and NO generation is evidenced by widespread nitrotyrosine (NT) staining in tumour tissue. Furthermore, presence of NT is observed in the majority of tumour-associated macrophages (TAMs), despite low iNOS expression by these cells, suggesting that NO from the tumour microenvironment affects TAMs. Indeed, using a co-culture model, we demonstrate that NO produced by colon and prostate cancer cells is sufficient to induce NT formation in neighbouring macrophages. Moreover, exposure to exogenous NO promotes mitochondria-dependent and -independent changes in macrophages, which orientate their polarity towards an enhanced pro-inflammatory phenotype, whilst decreasing antigen-presenting function and wound healing capacity. Abrogating endogenous NO generation in murine macrophages, on the other hand, decreases their pro-inflammatory phenotype. These results suggest that the presence of NO in cancer may regulate TAM metabolism and function, favouring the persistence of inflammation, impairing healing and subverting adaptive immunity responses.

## Highlights

• Nitric oxide (NO) from the cancer microenvironment acts on tumour-associated macrophages (TAMs).
• NO induces a phenotypic shift in macrophages through mitochondria-dependent and -independent pathways.
• NO favours pro-inflammatory cytokine production whilst decreasing macrophage function as antigen presenting cell.
• NO impairs repair function of macrophages.
• Presence of NO in cancer may regulate TAM metabolism and function, favouring tumour-promoting inflammation.

## Graphical abstract

**NO favours tumour-promoting inflammation by its actions on macrophages.** (1) NO within the tumour microenvironment acts on macrophages, leading to phenotypic alterations (2–4) that may favour the persistence of inflammation, impair healing and subvert adaptive immunity responses, thus contributing to tumour-promoting inflammation (created with BioRender.com).

**Figure:**
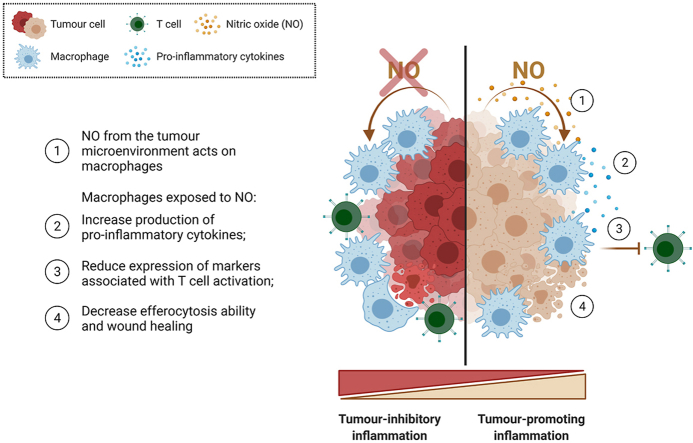

## 1 Introduction

Inflammation is associated with all stages of tumorigenesis and it can have both cancer-promoting and cancer-inhibitory effects [1,2]. Inflammation that favours tumorigenesis, mainly supported by innate immune cells such as macrophages [3], includes non-resolving, low-grade chronic inflammation [4], in which elevated levels of inflammatory mediators are maintained [4,5].

Increased production of the free radical nitric oxide (NO) by inducible nitric oxide synthase (iNOS, also named NOS2) [6] is associated with poor prognosis in several types of cancer. Previous histological studies in humans have demonstrated extensive iNOS staining in tumours. For instance, more than 90% of oestrogen receptor negative breast cancer patients die with moderate to high iNOS expression [7], and iNOS is found in approximately 80–100% of prostate cancer specimens [8], 60% of advanced melanoma patients [9], 60% of human colon adenomas and 20–25% of colon carcinomas [10], in contrast to normal tissue, in which low levels of iNOS are observed. Reaction products of NO such as peroxynitrite (ONOO^−^) have also been detected, in the form of nitrotyrosine (NT) [11], in a variety of cells within tumours [8,10,12], demonstrating the existence of a NO-rich microenvironment in cancer.

iNOS has been associated with the presence of inflammation in tumours, which either drive iNOS expression, or result from NO production [13,14]. Genetically engineered mice lacking iNOS (iNOS^−/-^ mice) or mice treated with iNOS inhibitors have reduced colon and gastric tumour formation, associated with decreased levels of pro-inflammatory cytokines produced by innate immune cells, such as TNF-α and IL-6, and diminished nitrosative and oxidative stress [[14], [15], [16]]. In human malignancies, iNOS co-expression with COX-2 is often observed [7,17]. On the other hand, NO has been shown to impede cellular immunity, and iNOS inhibitors improve efficiency of immunotherapy and radiotherapy in mice by enhancing T and NK cell infiltration and activity [[18], [19], [20], [21]]. The specific role of NO activity on macrophages, major drivers of tumour-associated inflammation, remains to be fully elucidated.

Here, we demonstrate iNOS expression and NO generation in biopsies of colon and prostate cancer. iNOS was mainly expressed by tumour cells, although it was also present in a low number of tumour-associated macrophages (TAMs). Using a co-culture model, we show that NO produced by colon and prostate cancer cells mediates its effects on neighbouring macrophages, leading to alterations in cytokine production. Furthermore, we found that NO, through mitochondria-dependent and -independent mechanisms, shifts the balance of macrophage responses towards an enhanced pro-inflammatory phenotype, in which antigen-presenting and wound healing functions are impaired. These results suggest that presence of NO in cancer may regulate TAM metabolism and function, contributing to non-resolving, tumour-promoting inflammation.

## 2 Results

### 2.1 NO from the tumour microenvironment acts on macrophages

**Fig. 1:**
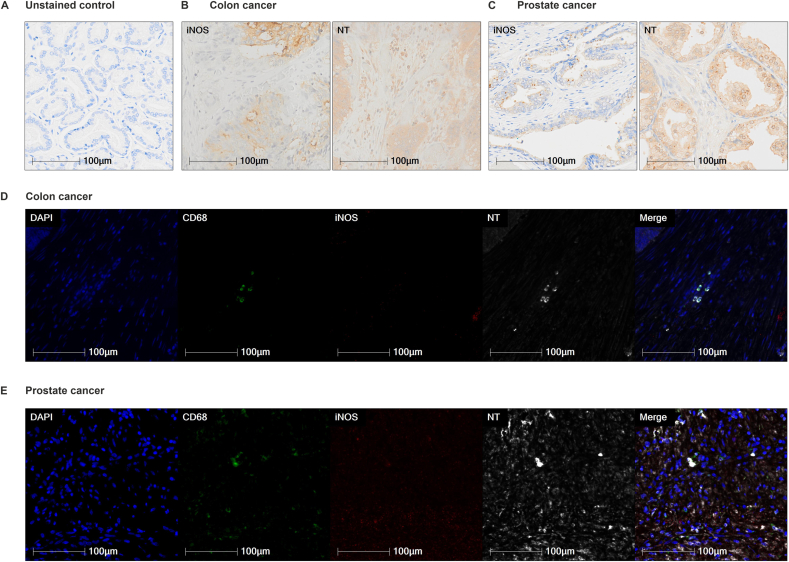
**- Expression of iNOS and NT in formalin-fixed, paraffin-embedded (FFPE) sections of human colorectal and prostate cancer.** Microphotograph of representative example of (**A**) unstained control, iNOS and NT expression in (**B**) colon (n = 2) and (**C**) prostate cancer (n = 5) tissue. (**D**) Microphotograph of representative example of multiplex immunofluorescence in colon and (**E**) prostate cancer samples using tyramide signal amplification (Perkin Elmer) for DAPI (blue), CD68 (green), iNOS (red), and NT (gray) staining. Images were acquired using the Olympus VS120 slide scanner at × 20 magnification. (For interpretation of the references to color in this figure legend, the reader is referred to the Web version of this article.)

We initially investigated whether iNOS activity is detected in human tumours. Expression of iNOS and nitrotyrosine (NT) were determined in tissue samples of surgically resected colorectal (n = 2, stage IV) and prostate (n = 5) (Supplementary Table 1) cancer by immunohistochemistry. For prostate samples, we also analysed areas of benign prostate hyperplasia (BPH), which positively associates with prostatic inflammation and later development of prostate cancer [22,23]. Macrophages were identified using the CD68 marker. iNOS and NT expression was found in all samples (Fig. 1A–C). iNOS was only present in a low number of TAMs, however >85% of macrophages in the tissue were positively stained for NT (Fig. 1D and E; Supplementary Figs. 1A and B). Similar results were observed in areas of BPH (data not shown).

To further investigate the presence of iNOS in tumours, we reanalysed publicly available single cell RNA-sequencing data of colorectal cancer [24]. Tumours (n = 7) were treatment-naïve, reflected different disease stages (I–IV) and were compared against matched non-malignant (normal) tissue. We observed that iNOS was mainly expressed by epithelial/cancer cells, and a higher number of iNOS^+^ epithelial cells was found in tumour than in surrounding areas (Supplementary Figs. 1C and D). Similar to our immunohistochemistry findings, iNOS was present at low levels in TAMs, and its expression was not significantly different from macrophages from non-malignant tissue (Supplementary Fig. 1D). However, amongst several differentially expressed genes (Supplementary Fig. 1E) and enriched pathways, TAMs (n = 1,535 cells) had significantly increased activation of NO-related signalling when compared to macrophages from normal areas (n = 642 cells), including protein nitrosylation and NO-mediated signal transduction (Supplementary Fig. 1F).

The observation that nitrotyrosine and NO signalling are increased in TAMs, despite low iNOS expression by these cells, suggests that NO from the tumour microenvironment mediates its effects on macrophages. To test this hypothesis, we developed prostate (PC3) and colon (HT-29) cancer cell lines expressing iNOS under the control of a tetracycline-inducible promoter (PC3-pLIX_403-hNOS2 and HT-29-pLIX_403-hNOS2, respectively) [25]. The cancer cells expressed iNOS and produced NO (measured by NO_2_^−^ formation by Griess assay) in a time- and dose-dependent manner following stimulation with doxycycline (Supplementary Figs. 2A,B,G,H). We then established a transwell co-culture system between cancer cells and human monocyte-derived macrophages, in which addition of doxycycline (5 μM) led to iNOS expression and NO production by cancer cells only (Supplementary Fig. 2C-E,I-K). Using this model, we observed that NO from the environment was able to act upon neighbouring cells, as evidenced by increased NT levels in macrophage lysates (Supplementary Fig. 2F,L), confirming that NO produced by tumour cells can mediate its effects in macrophages that do not express iNOS.

### 2.2 NO alters cytokine production by macrophages co-cultured with cancer cells

**Fig. 2:**
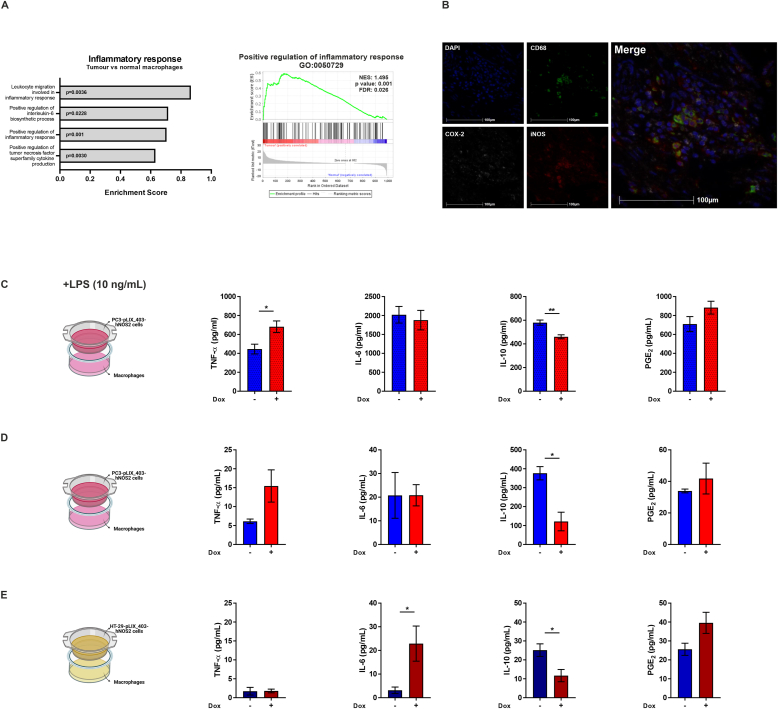
**- NO signalling associates with inflammation in tumours.** (**A**) Single-cell RNA-seq data of human colon cancer [24] were reanalysed with BBrowser2 (BioTuring). GO enrichment analysis of inflammatory pathways comparing tumour vs normal macrophages. The p value is indicated in the graph. A false discovery rate (FDR) q-value of less than 0.25 (25%) and p value < 0.05 obtained from BBrowser2 were considered statistically significant. GSEA of positive regulation of inflammatory response (GO:0050729) is shown. (**B**) Microphotograph of representative example of multiplex immunofluorescence in colon cancer samples using tyramide signal amplification (Perkin Elmer) for DAPI (blue), CD68 (green), iNOS (red), and COX-2 (gray) staining. Digital images of whole tissue sections were acquired using a Vectra scanner (Perkin Elmer) and images were analysed using the software HALO (Indica Labs). (**C**) Presence of TNF-α, IL-6, IL-10 and PGE_2_ was measured by ELISA from supernatants of macrophages co-cultured with PC3-pLIX_403-hNOS2 cells in the presence of LPS (10 ng/mL). (**D**) Presence of TNF-α, IL-6, IL-10 and PGE_2_ was measured by ELISA from supernatants of macrophages co-cultured with PC3-pLIX_403-hNOS2 cells. (**E**) Presence of TNF-α, IL-6, IL-10 and PGE_2_ was measured by ELISA from supernatants of macrophages co-cultured with HT-29-pLIX_403-hNOS2 cells. All values are mean ± SEM of at least three independent experiments. P values by unpaired *t*-test; *p < 0.05, **p < 0.01. (For interpretation of the references to color in this figure legend, the reader is referred to the Web version of this article.)

Production of NO in tumours has been positively associated with inflammation [13,14]. For instance, RNA-seq dataset reanalysis of colon tumours [24] demonstrated that TAMs, in which NO signalling was increased, also exhibited enhanced inflammatory pathway activation, including TNF-α and IL-6 signalling, when compared to macrophages from normal areas (Fig. 2A). Previous studies in cancer have also demonstrated an interaction between iNOS and COX-2, which participates in tumour-associated inflammation through generation of PGE_2_ [7,17]. Even though COX-2 was not differentially expressed between normal and tumour macrophages in the RNA-seq reanalysis (data not shown), presence of COX-2 in CD68^+^ cells expressing iNOS or near these populations was observed when we further stained FFPE blocks of colorectal cancer (Fig. 2B), supporting the hypothesis of a relationship between NO and inflammatory pathways in tumours.

To investigate this association further, we co-cultured macrophages with PC3-pLIX_403-hNOS2 prostate cancer cells. We stimulated macrophages towards an inflammatory phenotype with lipopolysaccharide (LPS) (10 ng/mL) and assessed whether presence of NO in the environment promotes alterations in cytokine secretion by macrophages. Exposure to NO caused a significant increase in TNF-α levels and decreased IL-10 secretion by cells, suggesting an enhancement of pro-inflammatory cytokine environment in the presence of NO (Fig. 2C). This effect was also observed, although to a lesser extent, in macrophages without any prior stimulation. Non-activated macrophages co-cultured with NO-producing PC3-pLIX_403-hNOS2 cells demonstrated a trend towards increased production of TNF-α, whereas IL-10 levels were reduced (Fig. 2D). Similarly, co-culture of non-stimulated macrophages with NO-producing colon cancer cells led to an increase in IL-6 levels and significantly decreased IL-10 secretion (Fig. 2E). These results suggest that NO generated by cancer cells regulates the responses of neighbouring macrophages, both activated and non-activated, favouring a pro-inflammatory environment. Thus, NO may alter the balance of cancer-associated inflammation.

### 2.3 NO enhances pro-inflammatory cytokine production by macrophages through mitochondria-dependent and -independent mechanisms

**Fig. 3:**
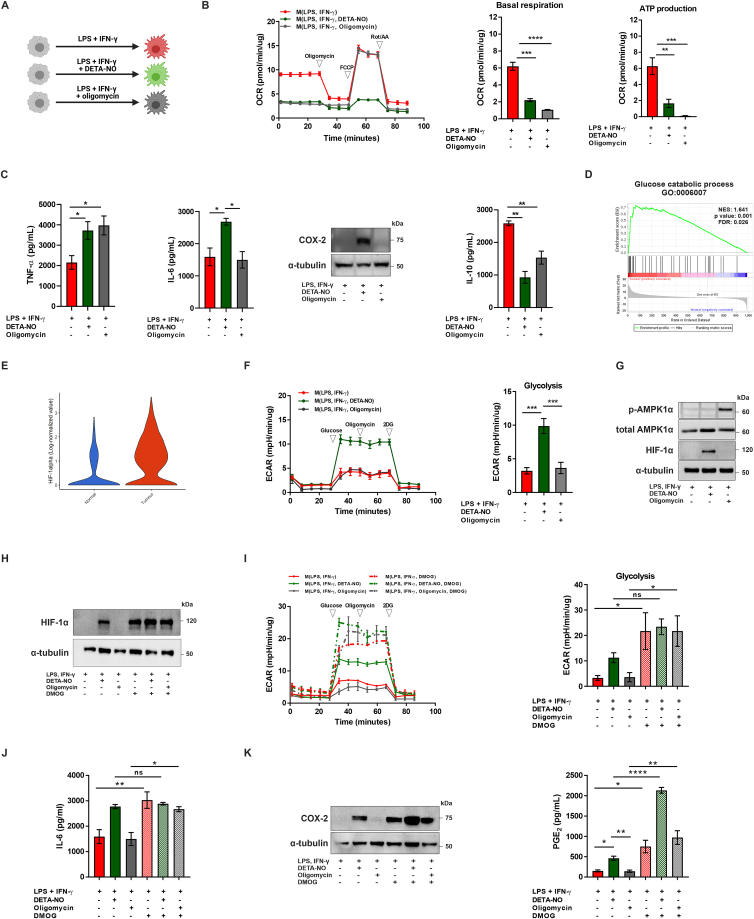
**NO enhances pro-inflammatory cytokine production by M1 macrophages through mitochondria-dependent and -independent mechanisms**. (**A**) Schematic diagram of human macrophage differentiation (created with BioRender.com). (**B**) Real-time changes in the oxygen consumption rate (OCR) of macrophages were assessed using a Seahorse XF Analyser after treatment with oligomycin, FCCP, and rotenone+antimycin A (Rot/AA) at the indicated time points. Basal respiration and ATP production were calculated according to the manufacturer’s instructions. (**C**) Production of TNF-α, IL-6, COX-2 expression and IL-10 secretion were assessed in human M1 macrophages following 24 h of activation. (**D**) Single-cell RNA-seq data of human colon cancer [24] were reanalysed with BBrowser2 (BioTuring). GSEA of glucose catabolic process (GO:0006007) and (**E**) HIF-1α expression were analysed in tumour vs normal macrophages. HIF-1α expression was significantly increased in tumour macrophages; p < 0.0001. (**F**) Real-time changes in the extracellular acidification rate (ECAR) were assessed after treatment with glucose, oligomycin, and 2-deoxyglucose (2-DG). Glycolysis was calculated according to the manufacturer’s instructions. (**G**) Analysis of phospho-AMPK (Thr172), total AMPK, HIF-1α and α-tubulin expression was assessed by western blotting after 6 h of macrophage activation. (**H**) Analysis of HIF-1α and α-tubulin expression in the presence of DMOG (1 mM) was assessed by western blotting after 6 h of treatment. (**I**) Real-time changes in the extracellular acidification rate (ECAR) were assessed in the presence of DMOG (1 mM). Glycolysis rate was calculated according to the manufacturer’s instructions. (**J**) Production of IL-6 was assessed by ELISA after 24 h. (**K**) COX-2 and α-tubulin expression were evaluated by western blotting after 24 h of treatment and PGE_2_ production was assessed by ELISA at the same time point. All values are mean ± SEM of at least three independent experiments. P values by one-way ANOVA followed by Tukey’s test; *p < 0.05, **p < 0.01, ***p < 0.001, ****p < 0.0001.

Macrophages can be polarised towards distinct functional phenotypes [26]. In order to understand the role of NO on different populations of macrophages, we next derived human M1 macrophages using LPS (10 ng/mL) and interferon (IFN)-γ (20 ng/mL), in the presence or absence of the slow-releasing NO donor (Z)-1-[2-aminoethyl]-N-(2-ammonioethyl)amino]diazen-1-ium 1,2-diolate (DETA-NO) (1 mM) (Fig. 3A). NO inhibits cellular respiration, including in murine M1 macrophages [27,28], therefore we first examined the impact of DETA-NO on oxygen consumption rate (OCR) as a measure of oxidative phosphorylation (OXPHOS). We observed that DETA-NO inhibited OXPHOS in human M1 macrophages, at 21% of oxygen, in a time- (Supplementary Figs. 3A–C) and dose-dependent manner (Supplementary Figs. 3D–E). After 24 h of activation, basal respiration and ATP production from OXPHOS were significantly reduced following DETA-NO treatment at the concentration of 1 mM (Fig. 3B). Because DETA-NO at this concentration can promote effects beyond inhibition of respiration, we also treated M1 macrophages with the ATP synthase inhibitor oligomycin (1 μM) (Fig. 3A), or with the inhibitors of electron transport chain (ETC) rotenone (1 μM) and antimycin A (1 μM), in order to distinguish the effects of NO that are independent of inhibition of respiration in mitochondria.

We then evaluated changes in cytokine production by cells. Exposure to NO led to a significant increase in secretion of the pro-inflammatory cytokines TNF-α and IL-6, and enhanced COX-2 expression. Conversely, production of IL-10 was decreased in macrophages treated with DETA-NO. These effects were observed after 24 h of activation (Fig. 3C) and were maintained after 120 h of NO exposure (Supplementary Fig. 4). Inhibition of respiration by oligomycin (Fig. 3B,C) as well as ETC inhibitors (Supplementary Figs. 5A and B) replicated the effects of NO on TNF-α and IL-10 secretion, demonstrating that these alterations are dependent on abrogation of respiration in mitochondria. Inhibition of respiration alone, however, did not alter the production of IL-6 and expression of COX-2, indicating that NO enhances IL-6 and COX-2 levels by mitochondria-independent mechanisms.

Previous studies have demonstrated that IL-6 production and COX-2 expression by macrophages can be regulated by glycolysis and HIF-1α signalling [[29], [30], [31], [32]]. Furthermore, both glycolytic pathway utilisation and HIF-1α can be altered by NO [33,34] and were shown to be increased in macrophages within colon tumours in the RNA-seq reanalysis [24] (Fig. 3D and E). We therefore assessed the impact of NO on glycolysis by measuring changes in the extracellular acidification rate (ECAR) of cultured macrophages. M1 macrophages enhanced glycolysis compared to non-activated cells (data not shown), as previously reported [35,36], and addition of DETA-NO promoted a further increase in the glycolytic pathway utilisation (Fig. 3F). This effect of NO was not replicated in M1 macrophages treated with oligomycin or inhibitors of ETC (Fig. 3F, Supplementary Fig. 6). Because NO can increase glycolysis by a mechanism dependent on AMP protein kinase (AMPK) pathway in certain cell types [34], we examined AMPK-Thr172 protein levels in macrophages (Fig. 3G). Treatment with NO, however, did not enhance AMPK phosphorylation, suggesting that NO was able to further enhance glycolysis in an AMPK-independent manner.

Next, we investigated the effects of NO treatment on HIF-1α stabilisation. NO but not oligomycin was able to increase HIF-1α protein levels in M1 macrophages (Fig. 3G). We used the prolyl hydroxylase inhibitor dimethyloxalylglycine (DMOG) (1 mM), which drives increased HIF-1α levels (Fig. 3H), to evaluate whether mitochondria-independent effects of NO are related to HIF-1α signalling. DMOG enhanced glycolytic pathway utilisation (Fig. 3I) and IL-6 production by macrophages (Fig. 3J), mimicking the effects of DETA-NO. Moreover, HIF-1α synergised with NO to increase COX-2 expression and PGE_2_ secretion, as PGE_2_ levels were approximately 4.8 and 2.8 fold enhanced after combination of DETA-NO and DMOG, compared to DETA-NO and DMOG alone, respectively (Fig. 3K). This demonstrates that the mitochondria-independent effects of NO are not only replicated by HIF-1α stabilisation, but NO may also synergise with HIF-1α in an inflammatory environment.

**Fig. 4:**
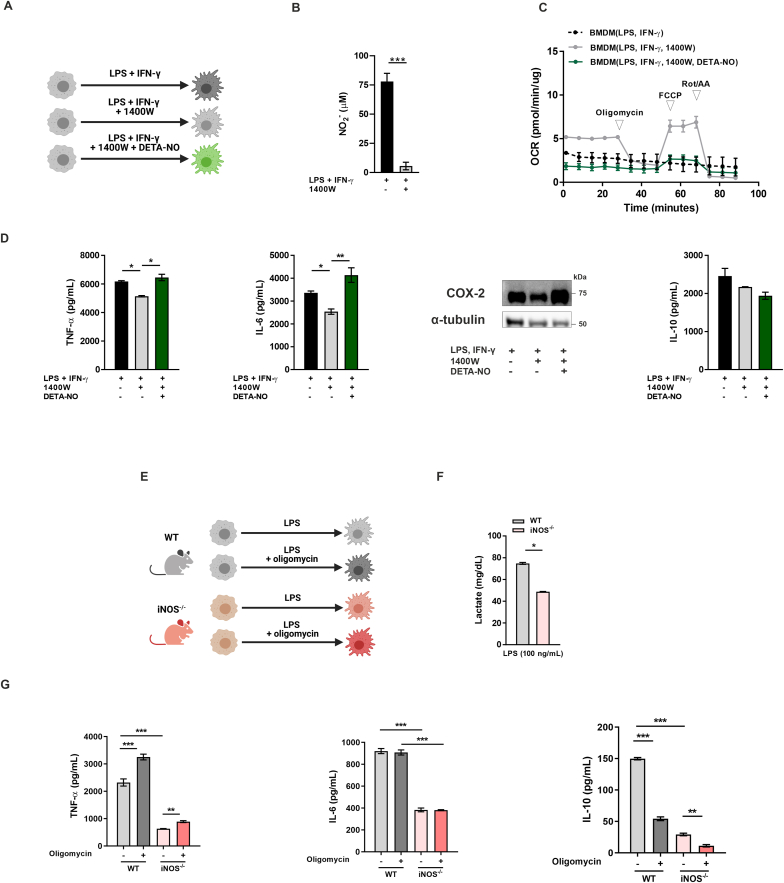
**– Endogenous NO enhances pro-inflammatory cytokine production by murine M1 macrophages.** (**A**) Schematic diagram of murine macrophage differentiation (created with BioRender.com). (**B**) NO_2_^−^ production by BMDMs was measured by Griess assay. (**C**) Real-time changes in the oxygen consumption rate (OCR) of macrophages were assessed using a Seahorse XF Analyser after treatment with oligomycin, FCCP, and rotenone+antimycin A (Rot/AA) at the indicated time points. (**D**) Production of TNF-α, IL-6, COX-2 expression and IL-10 secretion were measured following 24 h of activation. (**E**) Schematic diagram of murine macrophage differentiation from WT and iNOS^−/-^ B6 mice (created with BioRender.com). (**F**) Production of lactate, (**G**) TNF-α, IL-6 and IL-10 was assessed in M1 BMDMs from WT and iNOS^−/-^ mice. TNF-α, IL-6 and IL-10 secretion were assessed by ELISA following 24 h of activation in M1 macrophages from WT and iNOS^−/-^ mice treated with oligomycin (5 μM). All values are mean ± SEM of at least three independent experiments. P values by one-way ANOVA followed by Tukey’s test or unpaired *t*-test; *p < 0.05, **p < 0.01, ***p < 0.001.

As these results were obtained with addition of exogenous NO, we also examined whether endogenously-generated NO promotes the same effects on cytokine secretion. For that purpose, we abrogated iNOS expression in murine macrophages by pharmacological blockade and genetic knockout of the enzyme. Murine bone marrow derived macrophages (BMDMs) differentiated towards M1 were treated with the iNOS inhibitor 1400W (100 μM) (Fig. 4A), which supressed NO production (Fig. 4B) as well as a mitochondrial dysfunction (Fig. 4C), as shown by others [37,38]. Cells treated with 1400W had significantly decreased TNF-α and IL-6 secretion as well as COX-2 expression when compared to vehicle, and a similar trend was observed for IL-10. Inhibition of respiration and cytokine levels were rescued by addition of exogenous NO (1 mM) (Fig. 4D). These results were also replicated with the murine monocyte/macrophage-like cell line Raw 264.7 (data not shown). We then conducted the experiments with BMDMs from iNOS^−/-^ mice (Fig. 4E). M1 macrophages lacking iNOS expression showed reduced lactate levels in the supernatant (Fig. 4F), suggesting decreased glycolytic metabolism in the absence of NO. TNF-α, IL-6 and IL-10 secretion was also reduced when compared to WT mice. Mitochondrial dysfunction induced by oligomycin elevated TNF-α production and reduced IL-10 levels in both WT and iNOS^−/-^ mice, but no changes in IL-6 production were observed (Fig. 4G). Thus, these data indicate that endogenously-generated NO has similar effects as exogenous NO on macrophage cytokine secretion, and such effects can be partially replicated by inhibition of respiration with oligomycin.

We next examined whether the effects of NO are maintained when macrophages are incubated with other stimuli such as zymosan A (*Saccharomyces cerevisiae*) and poly(I:C), which bind to TLR2 and TLR3, respectively (Supplementary Fig. 7A) [39]. Addition of DETA-NO (1 mM) promoted similar increases as described before for LPS and IFN-γ in response to zymosan A (Supplementary Fig. 7B) and poly(I:C) (Supplementary Fig. 7C), demonstrating that NO actions are independent of TLR4 signalling and conserved between different classes of pathogens. Together, our results strongly suggest that M1, pro-inflammatory function of macrophages is enhanced in the presence of exogenous or endogenous NO, irrespective of the stimuli inducing inflammation, by mechanisms that are dependent and independent of inhibition of respiration in mitochondria.

### 2.4 NO decreases macrophage function as antigen presenting cell

**Fig. 5:**
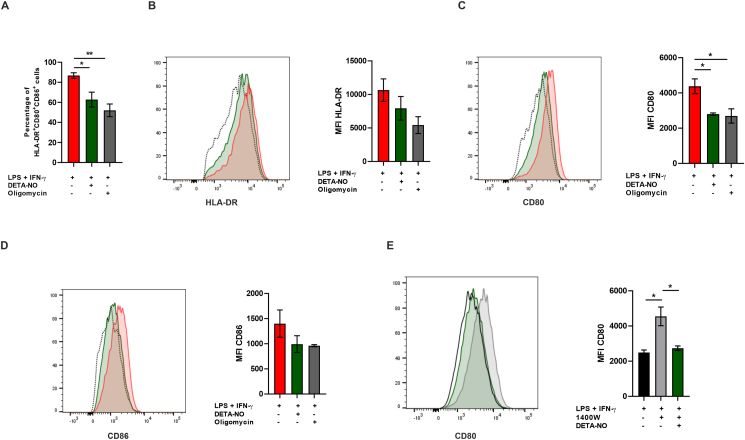
**- NO decreases expression of molecules required for antigen presentation.** Human M1 macrophages activated for 6 h were stained for HLA-DR, CD80 and CD86. (**A**) Percentage of HLA-DR^+^CD80^+^CD86^+^ cells was assessed by flow cytometry. FMO controls were used for gating positive populations. Median fluorescence intensity (MFI) was calculated for (**B**) HLA-DR, (**C**) CD80 and (**D**) CD86. Representative histograms are shown. (**E**) Murine BMDMs were stained for CD80 after 24 h of activation. Median fluorescence intensity (MFI) and representative histogram is indicated. All values are mean ± SEM of at least three independent experiments. P values by one-way ANOVA followed by Tukey’s test; *p < 0.05, **p < 0.01.

Macrophages function as antigen presenting cells (APCs) through interaction of peptide loaded MHC with the T cell receptor (TCR). Macrophages also provide co-stimulatory signals to T cells via CD80/CD86 binding to CD28 on the T cell [40], promoting their activation and expansion. Decreased expression of MHC, CD80 and CD86 on APCs correlates with impaired T cell responses and immunosuppression in cancer [41]. To understand the role of NO on macrophage antigen presenting function, we assessed the expression of HLA-DR (MHC class II), CD80 and CD86 on human M1 macrophages by flow cytometry. DETA-NO (1 mM) and oligomycin (1 μM) led to a reduction in the percentage of cells co-expressing all three molecules (Fig. 5A) and NO promoted a significant decrease in the median fluorescence intensity (MFI) of CD80 (Fig. 5B–D). These changes were observed at early stages of macrophage differentiation (6 h). Murine M1 macrophages expressing iNOS or exposed to DETA-NO presented similar levels of CD80 expression after 6 h of activation, when compared to macrophages in which iNOS was inhibited (data not shown). However, CD80 expression was significantly higher after 24 h in the absence of NO (Fig. 5E). These results indicate that exogenous and endogenous NO decrease macrophage-derived molecules important for T cell activation.

### 2.5 NO impairs wound healing function of macrophages

**Fig. 6:**
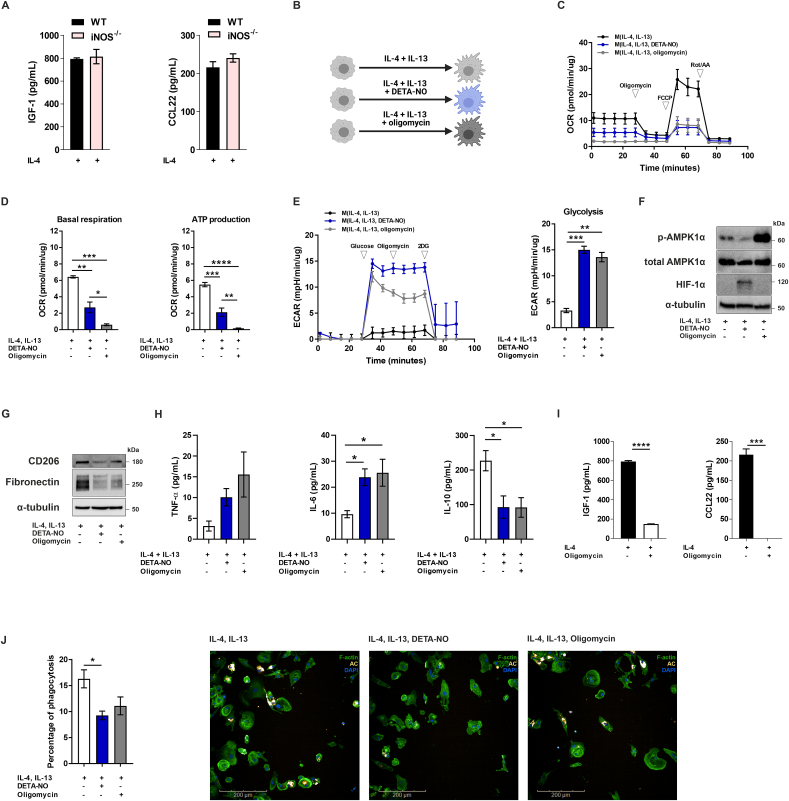
**- NO impairs resolution ability of macrophages**. (**A**) Production of M2 markers IGF-1 and CCL22 was assessed by ELISA in M2 BMDMs from WT and iNOS^−/-^ mice. (**B**) Schematic diagram of human macrophage differentiation (created with BioRender.com). (**C**) Real-time changes in the oxygen consumption rate (OCR) of macrophages were assessed using a Seahorse XF Analyser after treatment with oligomycin, FCCP, and rotenone+antimycin A (Rot/AA) at the indicated time points. (**D**) Basal respiration and ATP production were calculated according to the manufacturer’s instructions. (**E**) Real-time changes in the extracellular acidification rate (ECAR) were assessed after treatment with glucose, oligomycin, and 2-deoxyglucose (2-DG). Glycolysis was calculated according to the manufacturer’s instructions. (**F**) Analysis of phospho-AMPK (Thr172), total AMPK, HIF-1α and α-tubulin expression was assessed by western blotting. (**G**) Analysis of the M2 markers CD206 and fibronectin was assessed by western blotting. (**H**) Production of TNF-α, IL-6 and IL-10 by macrophages was assessed by ELISA. (**I**) Murine M2 macrophages treated with oligomycin (5 μM) were assessed for production of IGF-1 and CCL22. (**J**) Percentage of phagocytosis of apoptotic neutrophils by human M2 macrophages. Apoptotic neutrophils (16–24 h post isolation) were stained with CellTracker CM-Dil Dye (Thermo Fisher Scientific) according to the manufacturer’s protocol. Stained apoptotic cells were enumerated and added to macrophages at a ratio of 1 apoptotic cell to 3 macrophages. Challenged macrophages were co-cultured at 37 °C for 3 h in RPMI media without FBS. Cells were washed and resuspended in PBS containing 1% PFA, stained for F-actin and DAPI and imaged using an Operetta or Opera Phenix microscope. Images were analysed using the Harmony software. Representative images demonstrate F-actin (green), apoptotic neutrophils (orange) and DAPI (blue) staining. All values are mean ± SEM of at least three independent experiments. P values by one-way ANOVA followed by Tukey’s test or unpaired *t*-test; *p < 0.05, **p < 0.01, ***p < 0.001, ****p < 0.0001. (For interpretation of the references to color in this figure legend, the reader is referred to the Web version of this article.)

Tumours have been previously described as wounds that do not heal [42], and macrophages with roles in resolution of inflammation and wound healing, known as M2 macrophages, are abundant in cancer [43]. Activation towards a M2 phenotype *in vitro* with cytokines such as IL-4 does not elicit iNOS expression [44], and M2 macrophages from WT and iNOS^−/-^ mice do not differ, as secretion of IL-4-induced cytokines was unchanged in macrophages from WT and iNOS^−/-^ mice (Fig. 6A). However, as we observed that NO from the tumour microenvironment can act on macrophages despite absence of iNOS expression, we also examined the impact of exogenous NO on M2 cells.

Human macrophages treated with the M2 stimuli IL-4 (20 ng/mL) and IL-13 (20 ng/mL), in addition to DETA-NO (1 mM) or oligomycin (1 μM) (Fig. 6B), presented a mitochondrial dysfunction (Fig. 6C,D) and increased glycolysis (Fig. 6E) as shown before for M1 macrophages. NO-treated cells were able to increase HIF-1α levels, and increase in AMPK phosphorylation was only observed following oligomycin treatment (Fig. 6F). Inhibition of respiration promoted a decrease in expression of fibronectin and CD206 (Fig. 6G), markers of M2 differentiation and function such as extracellular matrix production [45] and uptake of apoptotic cells [46], respectively. Secretion of pro-inflammatory cytokines such as IL-6 were increased, and production of IL-10 was reduced in the presence of a mitochondrial impairment (Fig. 6H). Murine M2 macrophages also had diminished secretion of the M2 cytokines IGF-1 and CCL22 when respiration was inhibited by oligomycin (Fig. 6I). This suggests that mitochondrial impairment favours features of M1 macrophage-like cells, even in the presence of M2-like polarising conditions.

Reduced CD206 expression suggested an impairment of macrophage phagocytosis. Efferocytosis, the process of removing apoptotic cells, is a crucial step during resolution of inflammation and tissue repair, preventing secondary necrosis and stimulating macrophages to secrete anti-inflammatory cytokines [47]. To evaluate whether NO alters efferocytosis, we co-cultured M2 macrophages with labelled apoptotic neutrophils for 3 h (Supplementary Fig. 8), in the presence or absence of NO or oligomycin. We then quantified cells that had engulfed neutrophils to calculate the percentage of efferocytosis. Approximately 16% of M2 macrophages had phagocytised an apoptotic neutrophil after 3 h. This number diminished to 9% in the presence of NO, indicating a reduced efferocytic ability by macrophages (Fig. 6J). Thus, our results demonstrate that NO decreases effector functions characteristic of M2 macrophages.

## 3 Discussion

Macrophages are abundant cells in solid tumours, in which their numbers correlate with poor prognosis [43]. Tumour-associated macrophages (TAMs) comprise heterogeneous populations that co-express M1 and M2 signals [43]. Although histological studies suggest that predominance of M1-like macrophages, defined by expression of markers such as CD80 and CD86, associates with better outcomes in cancer, the overwhelming evidence suggests that pro-inflammatory cytokines and other inflammatory mediators characteristic of M1 cells are known to contribute to tumour promotion and progression [4,5]. For instance, expression of the inducible isoform of nitric oxide synthase (iNOS) is characteristic to a M1-like phenotype, however iNOS is generally described as immunosuppressive, and its presence in tumours correlates with poor prognosis and resistance to therapy in both mice and human studies [7,[18], [19], [20], [21],[48], [49], [50], [51]]. Here, we further demonstrate that macrophages producing or exposed to NO undergo mitochondria-dependent and -independent changes, shifting their effector function towards an enhanced pro-inflammatory phenotype, whilst decreasing their expression of markers associated with T cell activation, including CD80, and reducing wound healing responses.

iNOS expression by macrophages is known to occur during infection and inflammation in mice, but there is no strong evidence of NO production by iNOS in human macrophages *in vitro* or in acute inflammation [52,53]. Some studies show that human macrophages have a hypermethylation in the iNOS locus [52] and that these cells might have decreased availability of the essential cofactor tetrahydrobiopterin, hence have limited ability to generate NO [54]. Nevertheless, we, in this study, and others show that in different types of cancer, chronic infections or inflammatory diseases iNOS is expressed [9,10,[55], [56], [57], [58]] and active in human macrophages, as demonstrated by the presence of tyrosine nitration [8,10,12] or NO synthase activity [59]. Furthermore, macrophages can be affected by NO produced by neighbouring cells within the microenvironment [60]. Indeed, we have observed widespread nitrotyrosine staining in the tumour samples, notably in TAMs lacking iNOS expression, and in lysates of macrophages co-cultured *in vitro* with tumour cells producing NO. Moreover, reanalysis of single cell RNA-seq data of colon cancer [24] revealed that TAMs increased activation of NO-related pathways, including protein nitrosylation and NO-mediated signal transduction, as well as inflammatory cytokine signalling, such as IL-6 and TNF-α. We further observed that macrophages co-cultured with NO-producing tumour cells increased secretion of these cytokines, supporting the hypothesis that NO, irrespective of cell source, may regulate macrophage responses and shift the balance of cancer-associated inflammation. Importantly, this occurred in non-activated macrophages as well as in cells cultured in the presence of an inflammatory stimuli.

The role of NO on macrophage function is controversial [44,[61], [62], [63], [64]]. Our results show that NO, in concentrations beyond those that inhibit respiration, as is the case when iNOS is induced, enhances the M1 macrophage phenotype, evidenced by increased and sustained pro-inflammatory cytokine production. In order to distinguish between its mitochondria-dependent and -independent actions, we compared the effects of NO to those achieved with oligomycin, which in previous studies has shown to exacerbate pro-inflammatory cytokine production by macrophages [37,38]. Indeed, we demonstrate that inhibition of respiration caused by NO, oligomycin or inhibitors of ETC contributes to an enhanced pro-inflammatory phenotype, observed by increased TNF-α production. Moreover, inhibition of respiration reduced IL-10 levels in M1 macrophages. IL-10 is a potent inhibitor of TNF-α generation [65], thus decreased IL-10 synthesis may contribute to exacerbate TNF-α levels.

Nevertheless, inhibition of respiration alone did not replicate the actions of NO on the secretion of IL-6 and PGE_2_ by M1 cells. This may be attributed to HIF-1α stabilisation by NO, as pharmacological stabilisation of HIF-1α with DMOG also increased both IL-6 and PGE_2_ synthesis by macrophages. At physiologic concentrations, NO reduces HIF-1α stabilisation by inhibiting respiration and redirecting O_2_ to prolyl hydroxylases, which target HIF-1α for proteasomal degradation [66]. However, at high concentrations, as is the case in our study, NO enhances HIF-1α stabilisation by mechanisms dependent on free radical formation [33,67]. Importantly, NO can affect TCA cycle enzymes and metabolites in a HIF-1α-independent manner, for instance by inhibiting aconitase 2 and pyruvate dehydrogenase [27], which may also contribute to the effects of NO on macrophage cytokine secretion.

The importance of glycolysis on pro-inflammatory cytokine production is well established [36]. NO can activate glycolysis through changes in AMP-activated protein kinase (AMPK), as a consequence of a rise in AMP/ATP ratio [34], or by stabilisation of HIF-1α. Here, we demonstrate that NO enhanced glycolysis in M1 macrophages by the latter pathway, through mechanisms beyond inhibition of respiration, as this response was not replicated by oligomycin, rotenone and antimycin A. It has been proposed that phosphorylation and activation of AMPK is suppressed following LPS and IFN-γ stimulation [68,69], therefore AMPK may be unable to switch on glycolysis in M1 macrophages. These differential responses in glycolysis may also contribute to the discrepancies in the production of IL-6 and PGE_2_ between macrophages treated with NO and oligomycin. Importantly, this only occurred in M1 macrophages, since both NO and oligomycin enhanced glycolysis in non-activated and M2 cells.

As tumour progresses, macrophages differentiate towards a M2-like phenotype, cells that are often associated with resolution of inflammation and wound healing [43]. Mitochondrial function is essential to M2 cells [36], and it is well established that inhibitors of respiration impair M2 polarisation and responses, such as arginase activity and secretion of M2 cytokines [37,70]. We show that activation and function of M2 macrophages, including efferocytosis, were also significantly reduced in the presence of NO due to inhibition of respiration, since these effects were mimicked by oligomycin. Furthermore, production of pro-inflammatory cytokines such as IL-6 was also increased in M2 macrophages exposed to NO. These changes may generate dysfunctional M2 cells, contributing to persistent inflammation, especially when clearance of apoptotic cells is impaired, ultimately enhancing infiltration of myeloid cells in tumours and favouring cancer growth.

An important aspect of anti-tumour immunity is related to the presence and activity of tumour infiltrating lymphocytes, which associate with better outcomes in several malignancies. We show that NO contributes to the maintenance of high concentrations of pro-inflammatory cytokines by macrophages and reduces their expression of markers related to T cell activation. Previous work has demonstrated that these factors possess a suppressive effect on T cell activity [71,72] and impair the ability of APCs to present antigen to T cells [61,73], respectively. Furthermore, there is evidence that nitration of proteins such as CCL2 and LCK decreases the number of tumour-infiltrating T cells in colon cancer [74] and it skews them towards an unresponsive phenotype in prostate cancer [75,76]. These findings strongly suggest that NO may subvert adaptive immunity responses, thus favouring tumour-promoting inflammation in cancer. Indeed, in pre-clinical models of cancer, iNOS inhibitors improve efficacy of radiotherapy and immunotherapy by enhancing lymphocyte infiltration and activity [[18], [19], [20], [21]].

We have also shown that NO increases both COX-2 and HIF-1α expression in macrophages *in vitro*. Similar to iNOS, COX-2/PGE_2_ and HIF-1α pathways have been suggested to favour tumour-promoting inflammation by mechanisms that include direct inhibition of cytotoxic cell infiltration and effector function [77,78]. S-nitrosylation of COX-2, a modification that enhances its catalytic activity, has been found in biopsies from colon and breast cancer patients [79]. Furthermore, co-expression of iNOS with COX-2 or HIF-1α is extensively reported in histological studies and associates with poor prognosis in several types of cancer [7,17]. iNOS, COX-2 and HIF-1α may act synergistically, and inhibition of iNOS may reduce both COX-2 and HIF-1α expression in tumours, enhancing the efficacy of cancer therapy [7,17,64,67].

Inflammatory responses in cancer are characterised by presence of both pro- and anti-inflammatory macrophage populations, ultimately shifting the balance towards immunosuppression and favouring tumour growth. We demonstrate that macrophages exposed to NO display an enhanced release of pro-inflammatory signals simultaneous with reduced wound healing function, whilst decreasing the expression of surface markers needed for T cell activation. As such, NO may act favouring the persistence of inflammation and subverting adaptive immunity responses, contributing to tumour development and progression.

## 4 Conclusion

Here, we show that NO, at a concentration beyond inhibition of cellular respiration, favours the maintenance of a pro-inflammatory environment by macrophages through mitochondria-dependent and -independent mechanisms. In addition, we show evidence that NO decreases APC function by macrophages, which may contribute to subvert adaptive immunity responses. These results suggest that NO production by iNOS is a factor contributing to non-resolving, tumour-promoting inflammation by inducing a phenotypic shift on macrophages.

## 5 Materials and methods

### 5.1 Histology and multiplexed immunofluorescence

Histology was performed on formalin-fixed paraffin embedded (FFPE) tissue; 4 μm sections were cut and mounted on charged slides. Dewaxing and heat induced epitope retrieval (HIER) of slides was automated on the Bond RX automated platform (Leica Microsystems) using epitope solution 1 (AR9961) for 20 min at 100 °C. Chromogenic IHC assays were performed with Bond Polymer Refine Detection kit (DS9800). Multiplex immunofluorescence staining was performed using the Open Research Kit (DS9777). Endogenous peroxidase was blocked using 3% hydrogen peroxide (VWR) for 10 min and the slides further blocked with 10% w/v casein (SP5020). Antibody application, detection and tyramide signal amplification (TSA) were conducted in sequential rounds. Primary antibodies were prepared in Bond antibody diluent (AR9352) and incubated for 30 min. These antibodies were anti-CD68 1:2,000 (Dako Omnis, Clone PG-M1), iNOS 1:400 (MAB9502), nitrotyrosine (NT) 1:200 (06–284 Upstate) and COX-2 1:400 (mAb #12282). Detection was performed using the appropriate secondary antibody (anti-mouse, Dako Envision System K4001 or anti-rabbit, Dako Envision System K4003) for 30 min, followed by TSA with PerkinElmer Opal 4-Color Automation IHC Kit (NEL800001KT) 1:200 for 10 min. Each antibody was then removed using a heat stripping step (epitope solution 1 (AR9961) for 10 min at 100 °C) and a new round of labelling was performed. Finally, nuclei were counterstained with DAPI (Thermo Fisher, 62248) for 15 min (0.33 μg/ml) and mounted in coverslips with ProLong Gold antifade mountant (Thermo Fisher, P36930). Antibody sequence and TSA-fluorophore selection were optimised to reduce non-specific staining. Bond antibody diluent was used as antibody substitute for negative control. Whole sections were then scanned using an Olympus VS120 slide scanner or Vectra scanner (Perkin Elmer) and images were analysed using the software HALO (Indica Labs) using the HighPlex FL module v3.2.1.

### 5.2 Single-cell RNA sequencing data analysis and Gene Set Enrichment Analysis (GSEA)

For single-cell RNA sequencing data analysis, BBrowser2 (Bioturing v2.2.4) software [80] was utilised to download colorectal cancer single-cell sequencing data [24]. The annotation of immune cell clusters and disease was based on the original cell clusters defined by the authors and downloaded within BBrowser2. Differential expression analysis was performed on BBrowser2 using the Venice method. Genes with p-values < 0.05 were considered as differentially expressed. The matrix of gene expression of macrophages was extracted using BBrowser2 for further gene set enrichment analysis (GSEA) [81] using the Broad Institute GSEA v2.07 software (http://www.broadinstitute.org/gsea), the molecular signatures database (http://www.broadinstitute.org/gsea/msigdb), and the C5: GO gene sets database. For GSEA analyses 1,000 phenotype permutations were performed in a weighted mode. A false discovery rate (FDR) q-value of less than 0.25 (25%) and p value < 0.05 obtained from BBrowser2 were considered statistically significant.

### 5.3 Isolation of human PBMCs from leukocyte cones and macrophage differentiation

Human blood from anonymous healthy donors was obtained in the form of leukocyte cones from the NHSBT Manchester Blood Centre (REC reference: 16/WM/0469). PBMCs were isolated using Ficoll Paque Plus (GE Healthcare). PBMCs were seeded in T175 flasks from Corning at a density of 100,000 cells/cm^2^ and monocytes were separated from lymphocytes by adherence to plastic. After 1 h of incubation, non-adherent cells were removed by two washes with phosphate-buffered saline (PBS). For differentiation to macrophages, monocytes were cultured in 30 mL of RPMI 1640 + GlutaMAX (Invitrogen) medium supplemented with 10% fetal bovine serum (FBS) (Gibco), 100 U of penicillin/mL (Sigma), 100 μg of streptomycin/mL (Sigma) and 50 ng/mL of M-CSF premium grade (Miltenyi Biotec) per flask for 7 days, at 37 °C and 5% CO_2_. In day 3, 10 mL of supplemented medium per flask was also added. In day 7, the macrophages were washed with PBS, and cells were removed with accutase (Sigma) and by gently scraping with a plastic cell scraper and PBS.

### 5.4 Macrophage culture and activation

Human macrophages were cultured with RPMI 1640 + GlutaMAX medium supplemented with 10% FBS, 100 U of penicillin/mL and 100 μg of streptomycin/mL. Macrophages were stimulated *in vitro* with 10 ng/mL of LPS (Sigma) and 20 ng/mL of IFN-γ (Peprotech); with 20 ng/mL of IL-4 (Peprotech) and 20 ng/mL of IL-13 (Peprotech); with 100 μg/mL of zymosan particles (Thermo Fisher Scientific); or with 1 μg/mL of poly(I:C) (Invivogen) for 24 h, unless stated otherwise. Macrophages were treated with the NO donor DETA-NO (Fisher Scientific) (100–1000 μM), antimycin A (1 μM), oligomycin (1 μM), rotenone (1 μM) and/or DMOG (1 mM).

### 5.5 Mice

8- to 12-week-old iNOS^−/−^ (B6.129P2-Nos2tm/Lau) mice and 8- to 12-week-old C57BL/6 WT mice (Harlan Olac Ltd) were used for bone marrow isolation. All animal procedures were approved by the Home Office UK and by the University of Manchester Animal Welfare and Ethical Review Body, and conformed to the requirements of the UK Animals (Scientific Procedures) Act, 1986.

### 5.6 Bone marrow derived macrophage (BMDM) culture and activation

Bone marrow cells from the femur of mice were isolated and cultured in RPMI-1640 with 2 mM l-glutamine, 10% fetal bovine serum (FBS), penicillin (100 U/mL), streptomycin (100 μg/mL) (Gibco), and 15% L929-conditioned medium or M-CSF. Macrophages were stimulated *in vitro* with 10 ng/mL of LPS (Sigma) and 20 ng/mL of IFN-γ (Peprotech); and with 20 ng/mL of IL-4 (Peprotech) and 20 ng/mL of IL-13 (Peprotech) for 24 h, unless stated otherwise. Macrophages were treated with the iNOS inhibitor 1400W dihydrochloride (Tocris Biotechne) (100 μM), NO donor DETA-NO (Fisher Scientific) (100–1000 μM) and/or oligomycin (1–5 μM).

### 5.7 Stable cell line generation

PC3 and HT-29 stable cell lines expressing iNOS under the control of tetracycline were generated by lentiviral transduction using pLIX403-hNOS2. pLIX403-hNOS2 was a gift from Edward Morgan (Addgene plasmid #110800; http://n2t.net/addgene:110800; RRID:Addgene_110800) [25]. Lentiviruses were produced in Lenti-X 293T cells seeded into 10 cm plates at 2 × 10^6^ cells per plate. For each plate of Lenti-X cells, a transfection mix comprising 3 μg of the transfer vector, 2 μg packaging plasmid, 1 μg envelope plasmid and 18 μg PEI was prepared in 1 mL OptiMEM and added dropwise to the cells. Cells were transfected for 16 h, after which the medium was replaced with fresh RPMI/10% FBS. Lentivirus-containing supernatant was collected after 24 h, replaced with fresh medium and collected again a further 24 h later. The supernatants were pooled, centrifuged at 1000×*g* for 5 min to remove cell debris, filtered through a 0.45 μm syringe filter, aliquoted and stored at −80 °C. PC3 and HT-29 cells (2 × 10^6^) were reverse-transduced by seeding into 10 cm plates in growth medium plus lentivirus-containing supernatant (1:5 ratio of virus:growth medium) and 8 μg/mL polybrene (Sigma #H9268). Cells were incubated with medium containing virus for 16 h, which was replaced with fresh growth medium to allow cells to recover for 24 h. Stable polyclonal cell lines were selected in 5 μg/mL puromycin for 14 days and expression of iNOS was confirmed by immunoblotting and Griess assay following doxycycline treatment.

### 5.8 Co-culture between PC3-pLIX_403 and HT-29-pLIX_403 cells and macrophages

The prostate cancer cell line (PC3) expressing iNOS under control of tetracycline (PC3-pLIX_403) was co-cultured with human macrophages for 120 h with or without doxycycline (5 μM), using 24 mm Transwell Multiple Well Plate with 0.4 μm pore PET membrane insert (Corning, cat number 10619141). The colon cancer cell line (HT-29-pLIX_403) was co-cultured with human macrophages for 96 h.

### 5.9 Nitrite and nitrotyrosine measurement

Supernatants were collected after different time points and stored at −80 °C until analysis. The Griess Reagent System (Promega) was used to measure nitrite (NO_2_^−^), according to manufacturer's instructions. Cell lysates were collected and levels of nitrotyrosine were measured by ELISA (ab210603) according to manufacturer's instructions.

### 5.10 Immunoblotting

Cells were harvested by scraping and washed with cold PBS, resuspended in 2x Lysis buffer (25 mM Tris, 6.4% glycerol, 0.8% SDS) and lysed by ultrasonication using a Soniprep 150 Plus (three times, 5 s each sonication, amplitude of 3 μm). The protein concentration was determined using the BCA Protein Assay Reagent (Thermo Scientific). 25–50 μg of the whole lysate was applied to SDS-PAGE and transferred to a nitrocellulose membrane (Perkin Elmer), followed by standard immunoblotting procedure. The bound primary antibodies were detected by the use of horseradish peroxidase (HRP)-conjugated secondary antibody and the ECL detection system (Clarity™ Western ECL Blotting Substrates, Bio-Rad, or SuperSignal™ West Dura Extended Duration Substrate, Thermo Scientific). Images were acquired in a ChemiDoc Touch Imaging System (Biorad).

### 5.11 Cytokine measurement

Supernatants were collected after different time points and stored at −80°C until analysis. Tumour necrosis factor (TNF)-α, interleukin (IL)-6, IL-10 (R&D Systems) and PGE_2_ (Enzo Life Sciences) levels were measured by ELISA according to manufacturer's instructions. Results were calculated according to manufacturer's instructions.

### 5.12 Extracellular acidification rate (ECAR) and the oxygen consumption rate (OCR) measurement

To analyse the extracellular acidification rate (ECAR) and the oxygen consumption rate (OCR), cells were analysed using a 96-well XF extracellular flux analyser (EFA) (Seahorse Bioscience). In brief, cells were plated (2 × 10^4^ cells/well in 80 μL) and either left unstimulated or stimulated as described. Cells were washed and analysed in XF Running Buffer (unbuffered stress test media; 2 mM glutamine in XF basal media, without FBS, pH 7.4, for glycolysis; and 2 mM sodium pyruvate, 2 mM glutamine and 10 mM glucose in XF basal media, without FBS, pH 7.4, for oxidative phosphorylation), according to the manufacturer’s instructions. Where indicated, ECAR was analysed in response to 10 mM glucose, 1 μM oligomycin and 100 mM 2-deoxyglucose (2-DG), and OCR was analysed in response to 1 μM oligomycin, 1 μM fluoro-carbonyl cyanide phenylhydrazone (FCCP) and 1 μM rotenone and 1 μM antimycin A. Results were normalised to μg of protein after measurement of protein concentration in each well.

### 5.13 Lactate measurement

Lactate levels in cell supernatants were quantified using a Lactate Colorimetric Assay Kit (Labtest), according to the manufacturer instructions.

### 5.14 Flow cytometry

Macrophages were blocked with 1% BSA in PBS for 15 min prior to antibody staining, or with Human TruStain FcX™ (Fc Receptor Blocking Solution, Biolegend). For detection of cell surface markers, antibodies were incubated with samples containing 2 × 10^5^ cells for 30 min. The following antibodies were used: anti-human CD86 (clone REA968) (Miltenyi Biotec), anti-human HLA-DR (clone L243), anti-human CD80 (clone 2D10), anti-mouse CD80 (clone 16-10A1), and anti-mouse F4/80 (clone BM8) from Biolegend. Following incubation, samples were washed and resuspended in PBS and 10,000–50,000 events recorded. All samples were acquired on a LSR II (BD Biosciences) flow cytometer and data was analysed using FlowJo software, version 10.

### 5.15 Neutrophil isolation and efferocytosis assay

Neutrophils were isolated from leukocyte cones by collecting granulocytes and red blood cell precipitate. Red blood cell lysis buffer (155 mM NH_4_Cl, 12 mM NaHCO_3_, 0.1 mM EDTA) addition followed by centrifugation were used to remove red blood cells. Neutrophils were then isolated using CD16 microbeads from Miltenyi Biotec (cat. number 130-045-701) according to manufacturer’s instructions. Apoptotic neutrophils (16–24 h post isolation) were stained with CellTracker CM-Dil Dye (Thermo Fisher Scientific) according to the manufacturer’s protocol. Stained apoptotic cells were enumerated and added to macrophages at a ratio of 1 apoptotic cells to 3 macrophages. Challenged macrophages were co-cultured at 37 °C for 3 h in RPMI media without FBS. Cells were washed and resuspended in PBS containing 1% PFA, stained for F-actin and DAPI and imaged using an Operetta or Opera Phenix microscope. Images were analysed using Harmony software.

### 5.16 Statistical analysis

Statistical procedures were performed in the Prism Version 8 software (GraphPad Prism) using Student's t-test or one-way analysis of variance (ANOVA) followed by Tukey’s post hoc analysis when appropriate. Unless stated otherwise, values represent the mean ± SEM of three replicates of one representative experiment (*p < 0.05, **p < 0.01, ***p < 0.001 and ****p < 0.0001).

## Author contributions

D.D. designed and conducted experiments, analysed data and wrote the manuscript. J.P.M.L. helped perform experiments and analyse data. C.A.S.H. analysed single-cell RNA-seq data. J.C.A.F., T.H., P.A.T. and S.M. contributed to the study design, provided reagents and expertise, supervised the study and wrote the manuscript. All co-authors reviewed the manuscript.

## Declaration of competing interest

The authors declare that they have no known competing financial interests or personal relationships that could have appeared to influence the work reported in this paper.

## Appendix A Supplementary data

**Supplementary Table 1****-** Demographic and clinicopathological features of cases of prostate cancer. **Supplementary Fig. 1 - NO signalling is enhanced in tumour associated macrophages.** (**A**) Tumour-associated macrophages are positively stained for nitrotyrosine (NT). Microphotograph of representative example of multiplex immunofluorescence cancer samples using tyramide signal amplification (Perkin Elmer) for DAPI (blue), CD68 (green), iNOS (red), and NT (gray) staining. Macrophages were identified with the marker CD68. (**B**) Quantitation of NT^+^ macrophages in colon (n = 2) and prostate cancer (n = 5) tissue. Images were analysed using the software HALO (Indica Labs). Percentage (mean ± SEM) of NT^+^ macrophages is shown. Each dot represents an individual patient. (**C**) Single-cell RNA-seq data of human colon cancer [24] were reanalysed with BBrowser2 (BioTuring). t-SNE plots show cell type composition and iNOS expression. (**D**) Violin plot for iNOS expression in epithelial cells and macrophages in normal and tumour areas. (**E**) Differentially expressed genes between tumour and normal macrophages. Differential expression analysis was performed on BBrowser2 using the Venice method. Genes with p-values < 0.05 were considered as differentially expressed. (**F**) GO enrichment analysis of nitric oxide related pathways comparing tumour vs normal macrophages. The p value is indicated in the graph. A false discovery rate (FDR) q-value of less than 0.25 (25%) and p value < 0.05 obtained from BBrowser2 were considered statistically significant. GSEA of protein nitrosylation (GO:0017014) and nitric oxide mediated signal transduction (GO:0007263) are shown. Normalized enrichment scores (NES) were obtained from GSEA software and are indicated in the graph. **Supplementary Fig. 2 - NO from the environment acts on macrophages that do not express iNOS.** (**A**) Prostate cancer (PC3) stable cell line expressing iNOS under the control of tetracycline was generated by lentiviral transduction using pLIX403-hNOS2 plasmid. Cells (PC3-pLIX_403-hNOS2) expressed iNOS in a dose-dependent manner following stimulation with doxycycline. (**B**) NO_2_^−^ production was assessed by Griess assay at different time points following addition of 5 μM of doxycycline in the media. (**C**) PC3-pLIX_403-hNOS2 cells were co-cultured with human macrophages for 120 h with or without doxycycline (5 μM), using a transwell system as shown in the schematic diagram (created with BioRender.com). (**D**) iNOS expression and (**E**) NO_2_^−^ production were assessed following 120 h of stimulation. (**F**) Presence of nitrotyrosine (NT) was measured in lysates from co-cultured macrophages by ELISA (Abcam). (**G**) Colon cancer (HT-29) stable cell line expressing iNOS under the control of tetracycline was generated by lentiviral transduction using pLIX403-hNOS2 plasmid. Cells (HT-29-pLIX_403-hNOS2) expressed iNOS following stimulation with doxycycline. (**H**) NO_2_^−^ production was assessed at different time points by Griess assay following addition of 5 μM of doxycycline in the media. (**I**) HT-29-pLIX_403-hNOS2 cells were co-cultured with human macrophages for 96 h with or without doxycycline (5 μM), using a transwell system as shown in the schematic diagram (created with BioRender.com). (**J**) iNOS expression and (**K**) NO_2_^−^ production were assessed following 96 h of stimulation. (**L**) Presence of nitrotyrosine (NT) was measured in lysates from co-cultured macrophages by ELISA (Abcam). All values are mean ± SEM. P values by unpaired *t*-test; *p < 0.05, **p < 0.01, ***p < 0.001. **Supplementary Fig. 3 - NO-induced inhibition of respiration in human M1 macrophages is time- and dose-dependent.** (**A**) NO_2_^−^ levels in supernatants were assessed by Griess assay following 6 h and 24 h of addition of DETA-NO to cells. (**B**) Real-time changes in the oxygen consumption rate (OCR) of M1 macrophages after 6 h and 24 h of activation. OCR was assessed by M1 macrophages treated with DETA-NO or oligomycin at the indicated time points. (**C**) Basal respiration, ATP production, and spare respiratory capacity were calculated according to the manufacturer’s instructions. (**D**) NO_2_^−^ levels in supernatants were assessed following addition of the indicated doses of DETA-NO to cells. (**E**) OCR, basal respiration and ATP production was compared between groups. All values are mean ± SEM of at least three independent experiments. P values by one-way ANOVA followed by Tukey’s test; *p < 0.05, **p < 0.01, ***p < 0.001, ****p < 0.0001. **Supplementary Figure 4 – NO maintains elevated pro-inflammatory cytokine levels by M1 macrophages.** Production of TNF-α, IL-6, IL-10 and PGE_2_ was assessed in human M1 macrophages by ELISA following 120 h of activation. All values are mean ± SEM of at least three independent experiments. P values by one-way ANOVA followed by Tukey’s test; *p < 0.05, **p < 0.01, ***p < 0.001. **Supplementary Fig. 5 - Effect of inhibition of respiration with rotenone and antimycin A on cytokine production by M1 macrophages.** (**A**) M1 macrophages were treated with antimycin A (AA) (1 μM) or rotenone (1 μM). Real-time changes in the oxygen consumption rate (OCR) of human macrophages were assessed using a Seahorse XF Analyser after treatment with oligomycin, FCCP, and rotenone+antimycin A (Rot/AA) at the indicated time points. (**B**) Production of TNF-α, IL-6, PGE_2_ and IL-10 was assessed by ELISA following 24 h of macrophage activation. All values are mean ± SEM of at least three independent experiments. P values by one-way ANOVA followed by Tukey’s test; *p < 0.05, **p < 0.01, ***p < 0.001. **Supplementary Figure 6 – NO, but not oligomycin and electron transport chain inhibitors, increase glycolysis in M1 macrophages.** Real-time changes in the extracellular acidification rate (ECAR) were assessed after treatment with glucose, oligomycin, and 2-deoxyglucose (2-DG) at the indicated time points in M1 macrophages treated with rotenone and antimycin A. Glycolysis was assessed in M1 macrophages following stimulation with DETA-NO (1 mM), oligomycin (1 μM), rotenone (1 μM) or antimycin A (1 μM) for 24 h. All values are mean ± SEM of at least three independent experiments. P values by one-way ANOVA followed by Tukey’s test; **p < 0.01, ***p < 0.001. **Supplementary Fig. 7 - NO enhances pro-inflammatory cytokine production independent of TLR4 signalling**. (A) Schematic diagram demonstrating activation of TLR signalling pathway in macrophages following stimulation with LPS, zymosan and poly(I:C) (created with BioRender.com). (B) Production of TNF-α, IL-6, and IL-10 was assessed in human macrophages by ELISA following 24 h of activation with zymosan (100 μg/mL). (C) Production of TNF-α, IL-6, and IL-10 was assessed in human macrophages by ELISA following 24 h of activation with poly(I:C) (1 μg/mL). All values are mean ± SEM of at least three independent experiments. P values by unpaired *t*-test; *p < 0.05, ***p < 0.001. **Supplementary Fig. 8 - Neutrophil isolation and induction of apoptosis.** Neutrophils were isolated from the granulocyte layer of leukocyte cones using CD16 microbeads (Miltenyi Biotec). (A) CD16^+^ cells were assessed for CD15 expression by flow cytometry. (B) Apoptosis was measured in neutrophils 16–24 h post isolation by flow cytometry. Dot plot graphs of unstained cells and annexin V (AV) and propidium iodide (PI) stained cells.
