# Supplementary material for: Nitric oxide favours tumour-promoting inflammation through mitochondria-dependent and -independent actions on macrophages
Source: Redox Biol. 2022 May 27;54:102350. doi: 10.1016/j.redox.2022.102350 (PMC9511697; doi:10.1016/j.redox.2022.102350)

Supplementary Table 1

PATIENT_NUMBER	Age at Surgery	PSA at surgery (ng/mL)	History/Diagnosis
C000218	64	5	Invasive adenocarcinoma
C003724	58	15	Prostatic adenocarcinoma
C004586	63	17	Acinar adenocarcinoma of prostate
C006526	57	9.24	Acinar adenocarcinoma<br>Gleason score: 4+3=7
C004920	64	6.2	Gleason 3 + 4 adenocarcinoma<br>pT3a acinar

Supplementary Figure 1

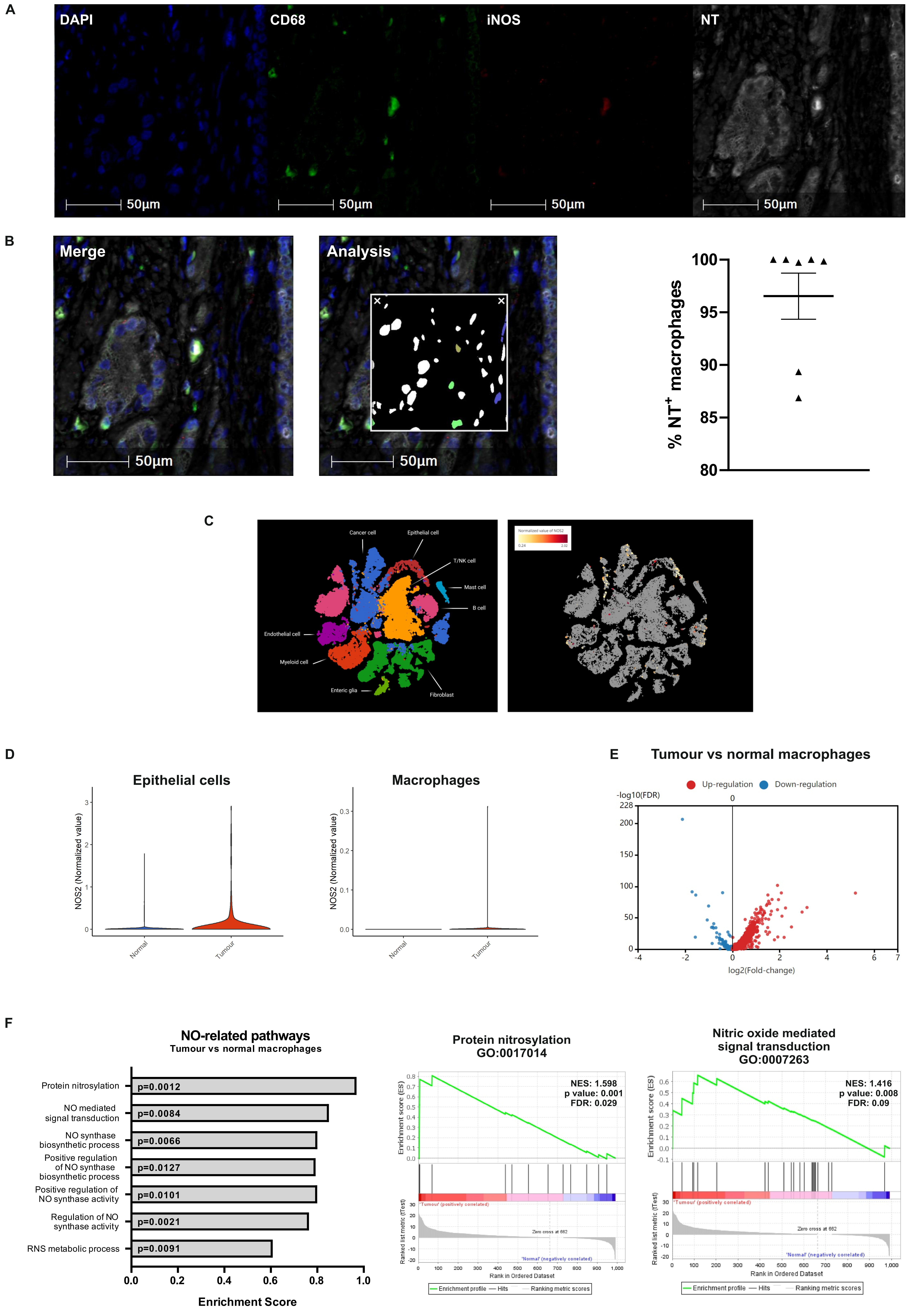

Supplementary Figure 2

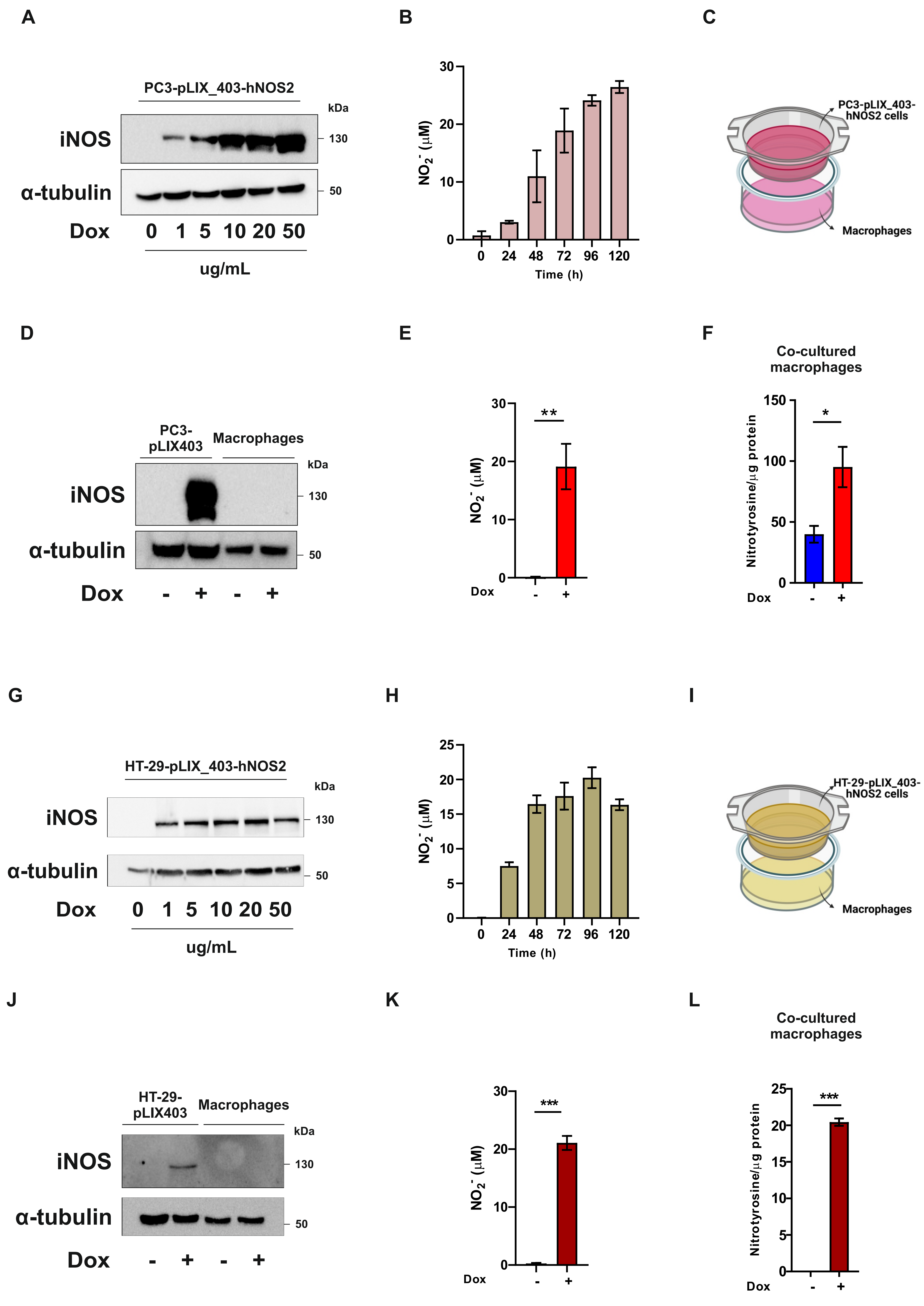

Supplementary Figure 3

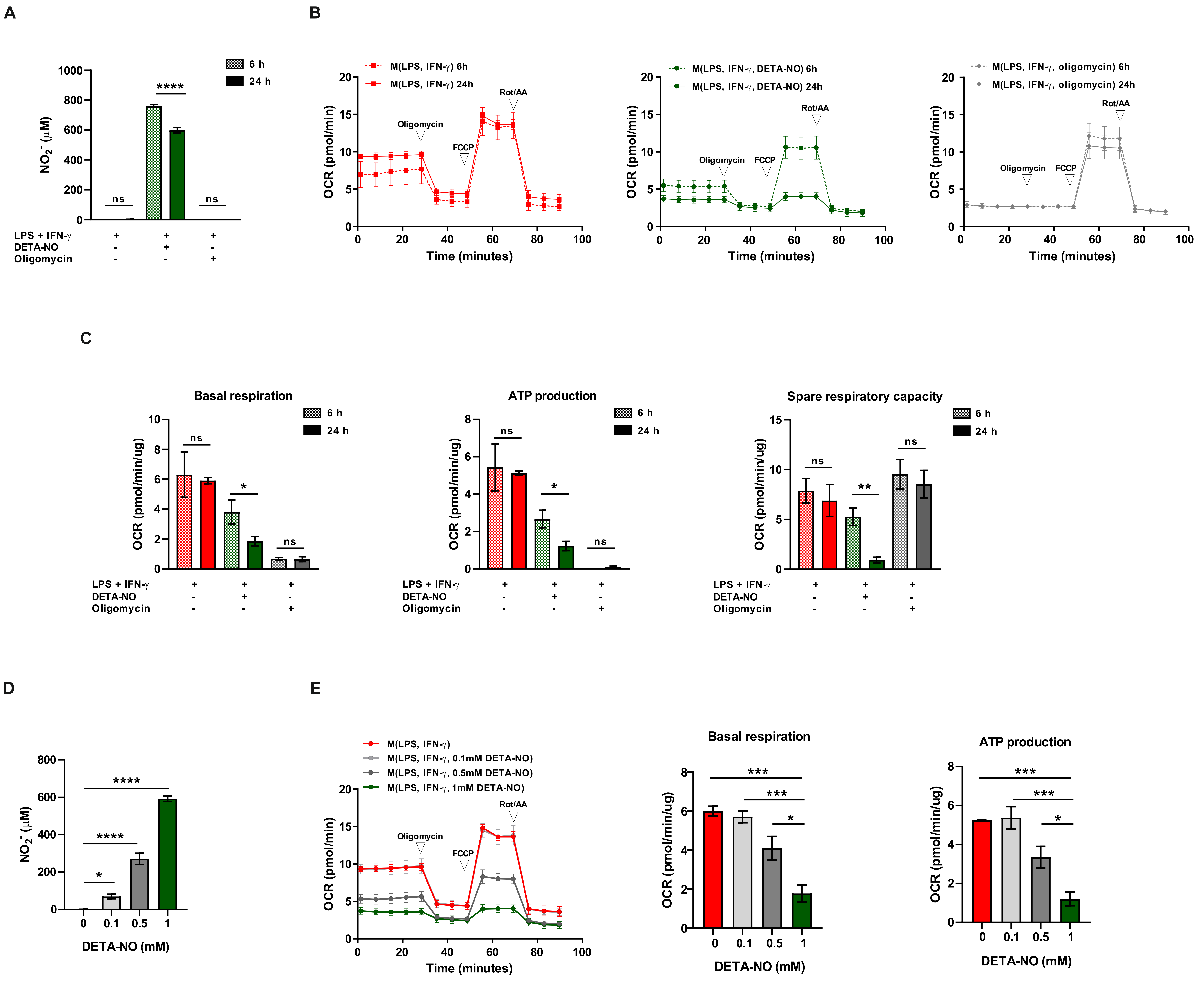

Supplementary Figure 4

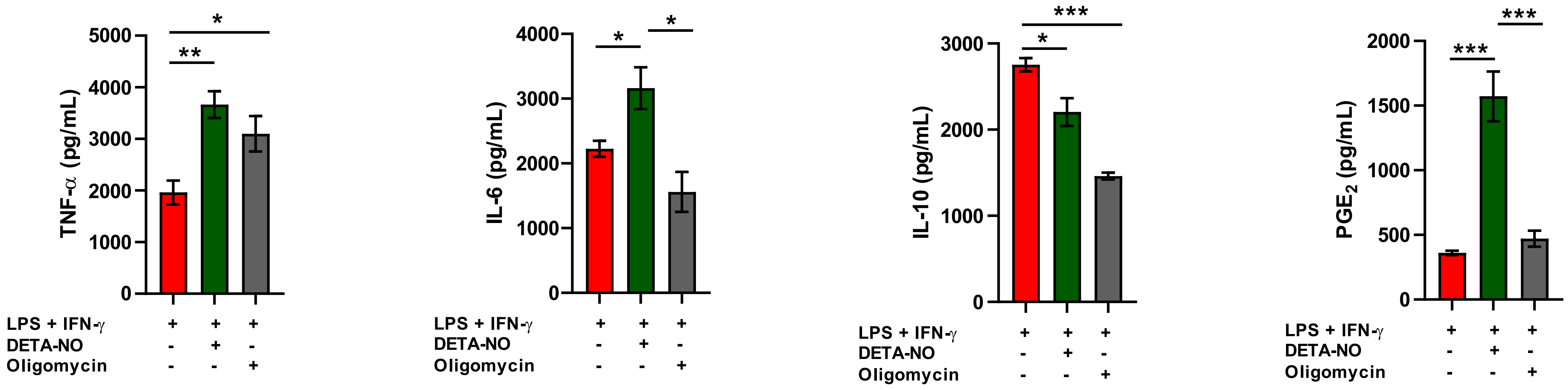

Supplementary Figure 5

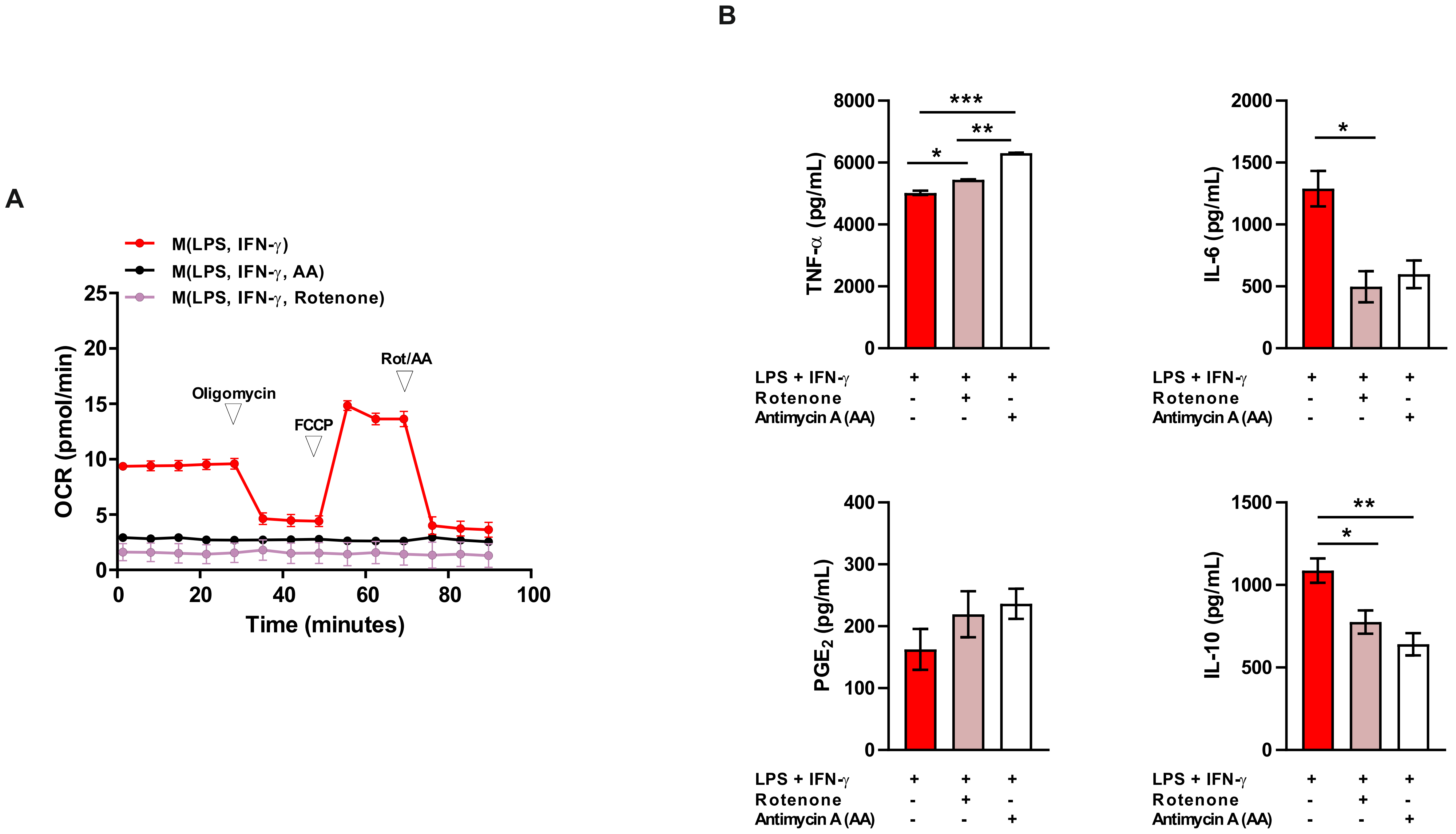

Supplementary Figure 6

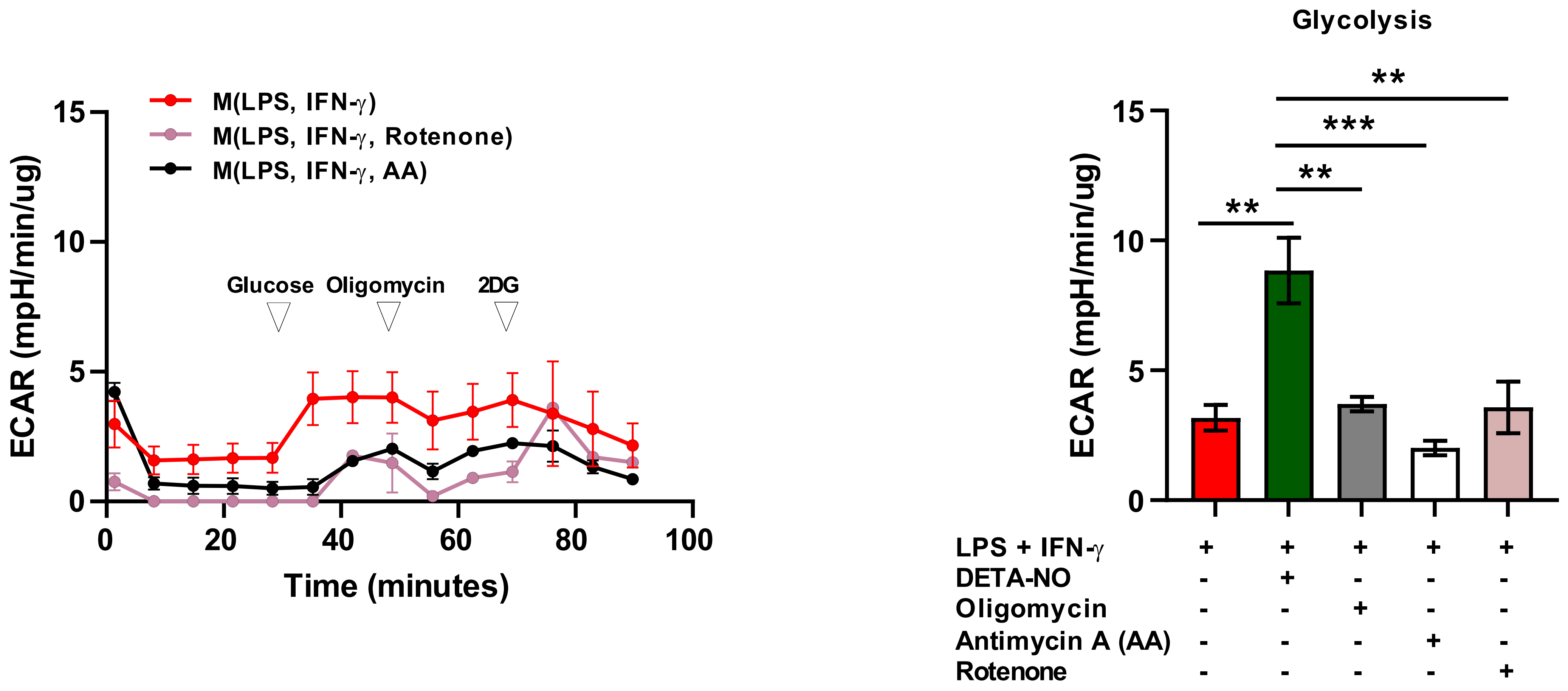

Supplementary Figure 7

A

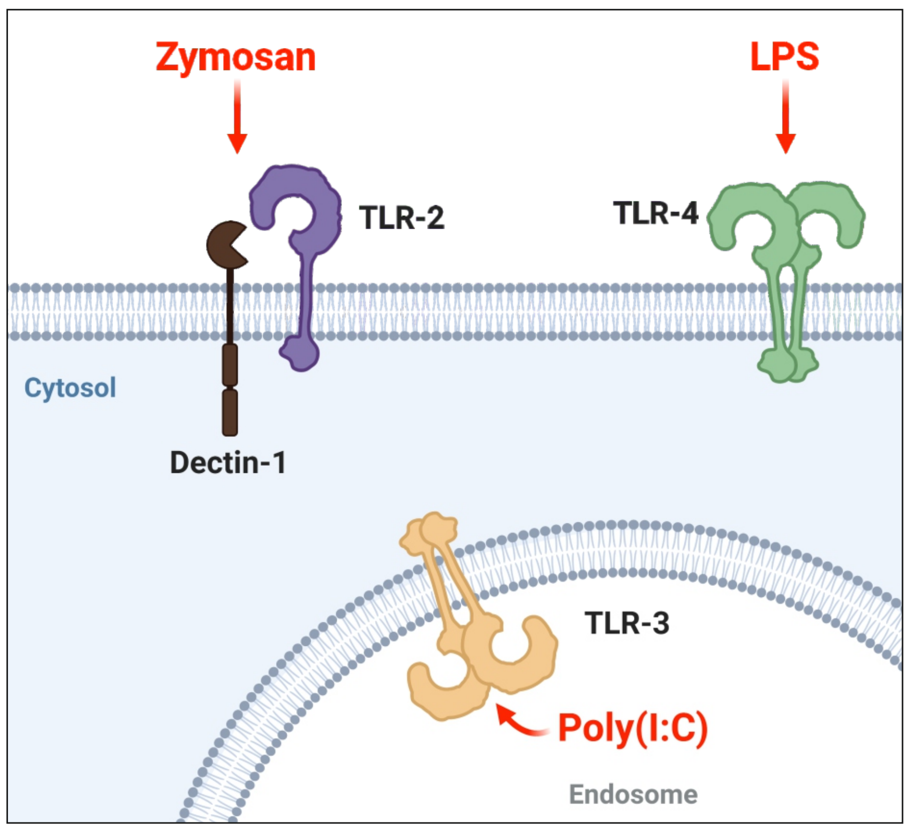

B

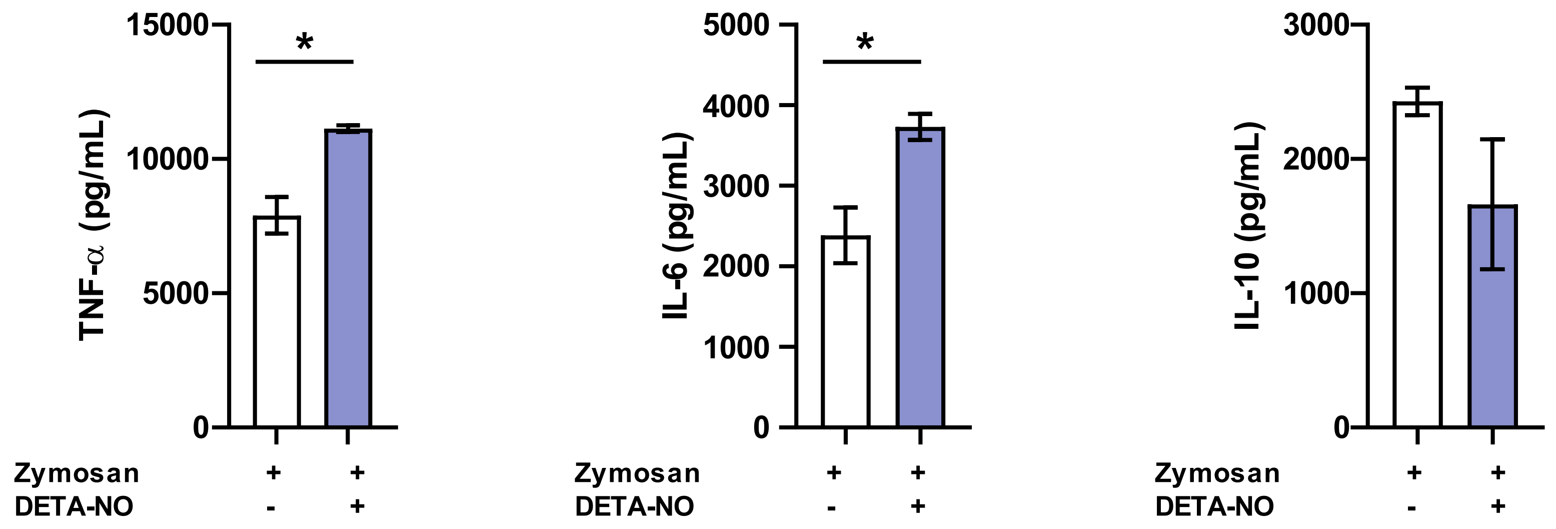

C

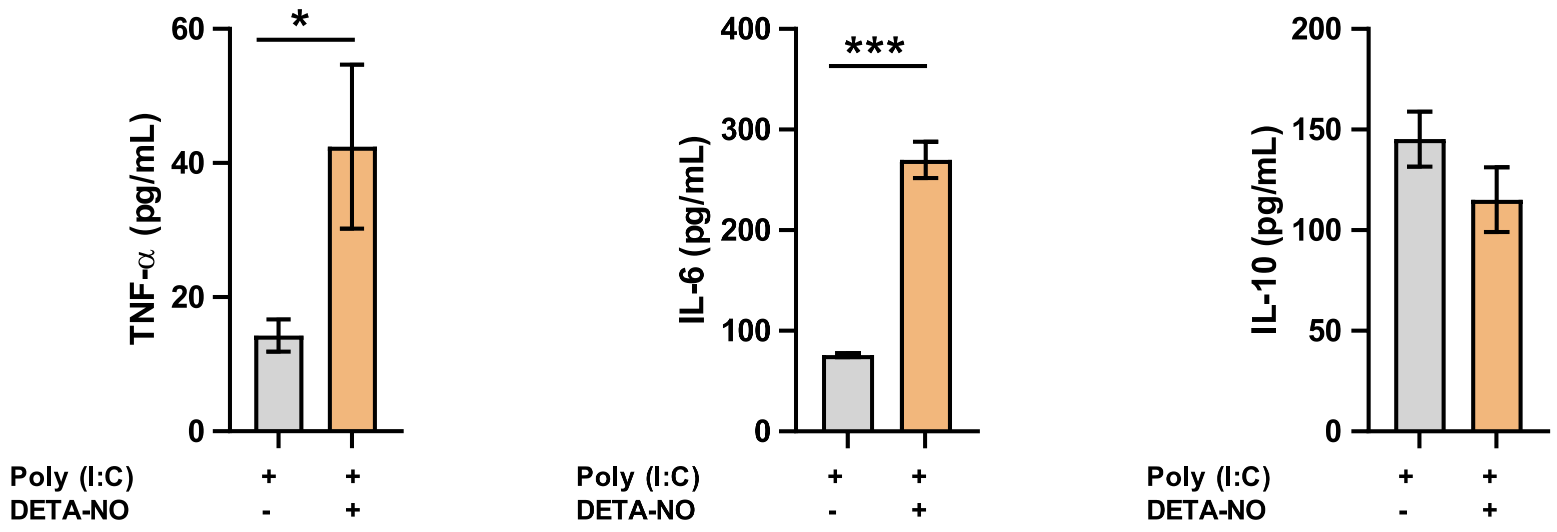

Supplementary Figure 8

A

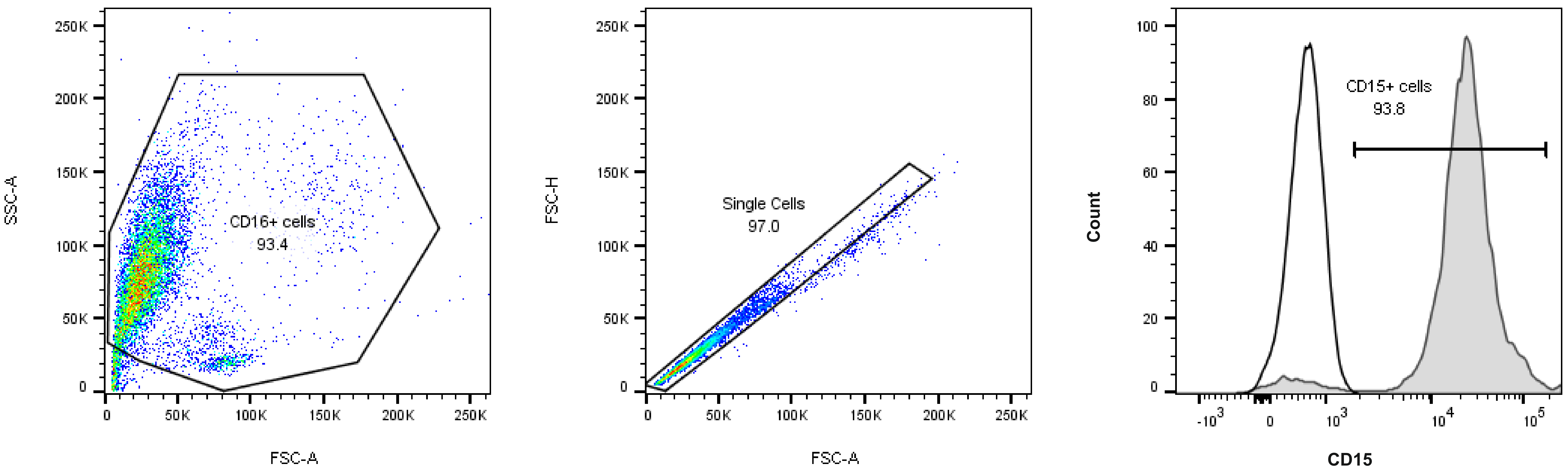

B

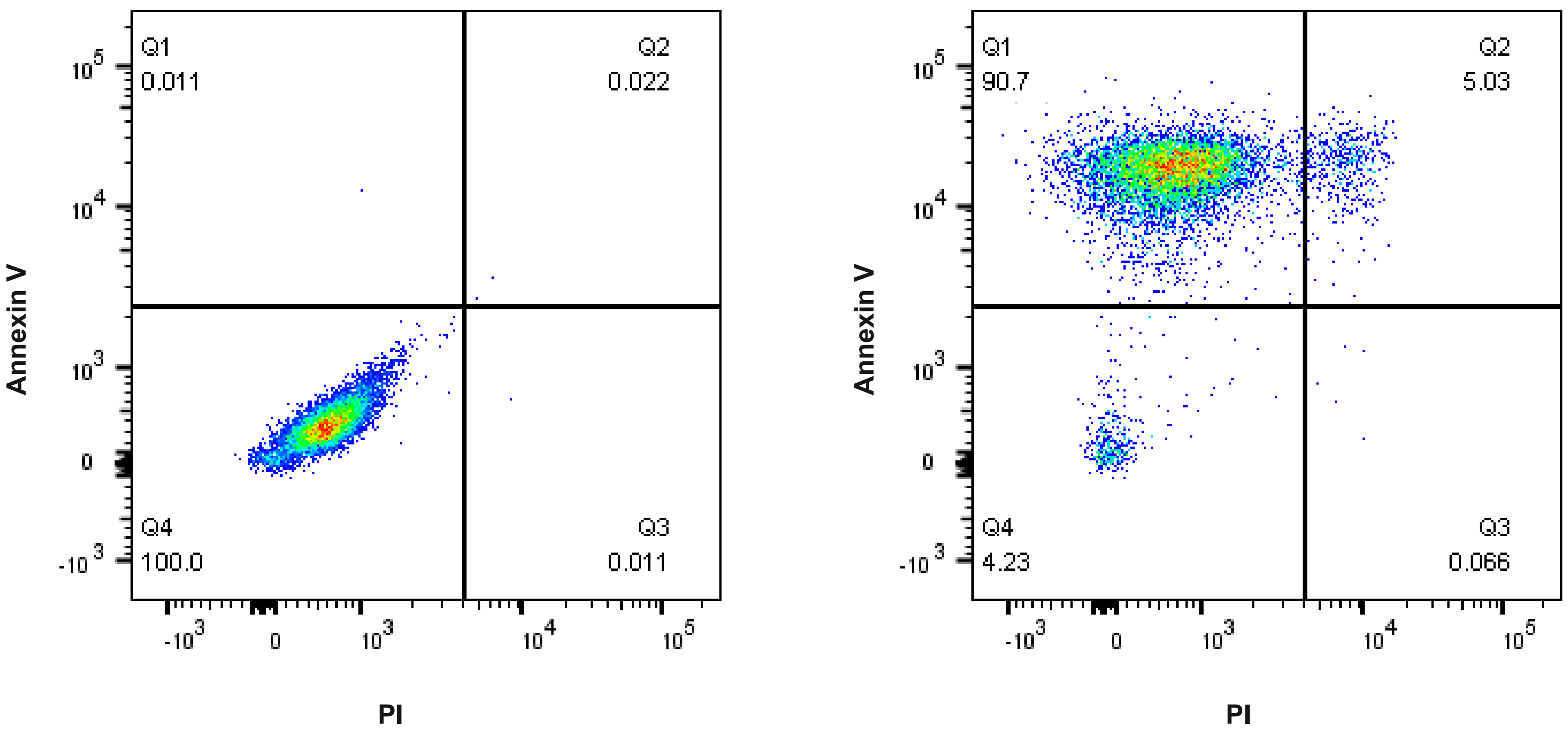

Supplement: Supplementary Figures [file mmc1.pdf]
